# Consanguinity and reproductive health among Arabs

**DOI:** 10.1186/1742-4755-6-17

**Published:** 2009-10-08

**Authors:** Ghazi O Tadmouri, Pratibha Nair, Tasneem Obeid, Mahmoud T Al Ali, Najib Al Khaja, Hanan A Hamamy

**Affiliations:** 1 Centre for Arab Genomic Studies, Dubai, United Arab Emirates; 2 Geneva Foundation for Medical Education and Research, Geneva, Switzerland

## Abstract

Consanguineous marriages have been practiced since the early existence of modern humans. Until now consanguinity is widely practiced in several global communities with variable rates depending on religion, culture, and geography. Arab populations have a long tradition of consanguinity due to socio-cultural factors. Many Arab countries display some of the highest rates of consanguineous marriages in the world, and specifically first cousin marriages which may reach 25-30% of all marriages. In some countries like Qatar, Yemen, and UAE, consanguinity rates are increasing in the current generation. Research among Arabs and worldwide has indicated that consanguinity could have an effect on some reproductive health parameters such as postnatal mortality and rates of congenital malformations. The association of consanguinity with other reproductive health parameters, such as fertility and fetal wastage, is controversial. The main impact of consanguinity, however, is an increase in the rate of homozygotes for autosomal recessive genetic disorders. Worldwide, known dominant disorders are more numerous than known recessive disorders. However, data on genetic disorders in Arab populations as extracted from the Catalogue of Transmission Genetics in Arabs (CTGA) database indicate a relative abundance of recessive disorders in the region that is clearly associated with the practice of consanguinity.

## Introduction

Linguistically, consanguinity is a term that is derived from two Latin words "*con*" meaning common, or of the same and "*sanguineus*" meaning blood, hence, referring to a relationship between two people who share a common ancestor or blood. In other words, consanguineous marriage refers to unions contracted between biologically-related individuals. In clinical genetics, a consanguineous marriage means union between couples who are related as second cousins or closer [1,2]. Among Arabs, this would include double first cousins, first cousins, first cousins once removed, and second cousins. Uncle-niece marriage is prohibited in Islam and so is absent among Arabs. In population genetics, consanguinity may also refer to unions of individuals with at least one common ancestor such as those occurring within population isolates, small towns, and tribes; intra-community or endogamous marriages. The custom of endogamy among individuals belonging to the same tribe (*hamula *or *kabeela*) is and has been strongly favored among Arabs, with the consequence of unequal distribution of founder mutations among the population. A large number of studies into the effects of consanguinity on health and disease have not taken such discrepancies into consideration.

## Consanguinity in World Populations

**Figure 1:**
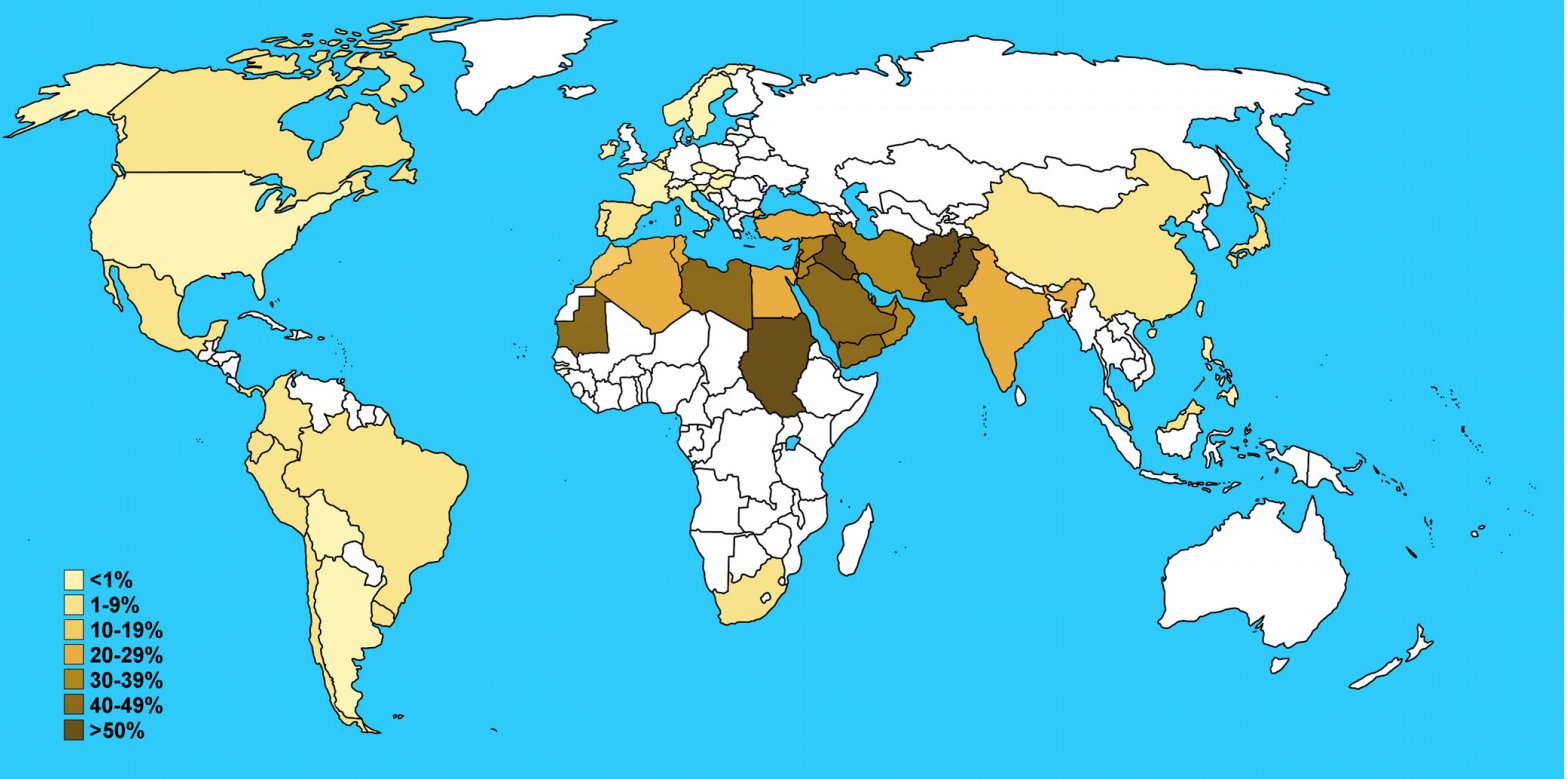
**Schematic representation of consanguineous marriage rates worldwide (adapted from Table 1, references **[82], **and **[139]. Only second-cousin and closer marriages are represented.

Consanguineous marriages have been practiced since the early existence of modern humans. At present, about 20% of world populations live in communities with a preference for consanguineous marriage [2]. Consanguinity rates vary from one population to another depending on religion, culture, and geography. Noticeably, many Arab countries display some of the highest rates of consanguineous marriages in the world ranging around 20-50% of all marriages, and specifically favoring first cousin marriages with average rates of about 20-30% (Table 1, Figure 1, Additional file 1).

**Table 1:** Consanguinity rates in Arab populations. Minimum and maximum reported rates are indicated when available

**Country**	**>1C, 1C**	**Overall consanguinity**	**References**
**Algeria**	11.3	22.6-34	[14,100]

**Bahrain**	24.5	39.4-45.5	[10,101]

**Egypt**	14.3-23.2	20.9-32.8	[15,70,102-104]

**Egypt (Nubia)**	39-47.2	60.5-80.4	[105,106]

**Iraq**	29-33	47-60	[86,107-109]

**Jordan**	19.5-39	28.5-63.7	[6,9,43,110-113]

**Kuwait**	16.9-31.7	22.5-64.3	[114-117]

**Lebanon**	6.7-31.6	12.8-42	[4,5,118-120]

**Libya**		48.4	[121]

**Mauritania**		47.2	[93]

**Morocco**	8.6-10	19.9-28	[21,122-124]

**Oman**	24.1	56.3	[125]

**Palestine**	13.6-34.2	17.5-66.3	[7,11-13,71,126-129]

**Qatar**	34.8	54	[19]

**Saudi Arabia**	24.6-42.3	42.1-66.7	[67,84,99,130,131]

**Sudan**	44.2-49.5	44.2-63.3	[66,132,133]

**Syria**	28.7	30-3-39.8	[16,134]

**Tunisia**	17.4-23	20.1-39.3	[18,9,135,136]

**United Arab Emirates**	20.7-28.2	40-54.2	[20,36,137]

**Yemen**	32-34	40-44.7	[17,138]

For comprehensive details and additional data, see Additional File 1.

Abbreviations: [>1C] = Double first-cousin marriage; [1C] = First-cousin marriage.

## Consanguinity in Arab Populations

Socio-cultural factors, such as maintenance of family structure and property, ease of marital arrangements, better relations with in-laws, and financial advantages relating to dowry seem to play a crucial role in the preference of consanguinity in Arab populations [3]. Consanguineous marriages are generally thought to be more stable than marriages between non-relatives, though there are no studies to compare divorce rates of consanguineous and non-consanguineous marriages among Arabs. It is generally believed that the husband's family would side with the consanguineous wife in marital disputes since she is considered part of the extended family. When there are children with disabilities, more family members share in caring for these children. Unlike what is thought, consanguinity in the Arab World is not only confined to Muslim communities. Several other communities, including the Lebanese, Jordanian, and Palestinian Christian populations, have also practiced consanguinity, but to a lesser extent than Muslims [4-7].

Consanguinity rates show wide variations among Arab countries, as well as within the same country (Table 1, Additional file 1). However, reports from Arab countries on consanguinity rates may sometimes include marriages between third cousins or far relatives within the consanguineous category. Although this discrepancy affects the total consanguinity rate, it does not markedly alter the average inbreeding coefficient. Therefore, for comparison of consanguinity rates among populations, two parameters are best used; the mean inbreeding coefficient (F) and marriages between first cousins. However, Arab societies have a long tradition of consanguinity, and the cumulative estimate of (F) may exceed the estimated value which is calculated for a single generation [8].

Secular changes in the consanguinity rates have been noticed in some Arab populations. In Jordan [9], Lebanon [5], Bahrain [10], and among Palestinians [11-13], the frequency of consanguineous marriage is decreasing. Several factors may be playing a role in decreasing the consanguinity rates in Arab countries. Amongst these factors are the increasing higher female education levels, the declining fertility resulting in lower numbers of suitable relatives to marry, more mobility from rural to urban settings, and the improving economic status of families. Moreover, genetic diseases may be feared more now that infectious diseases are on the decline as causes of severe morbidity and mortality.

Generally, the highest rates of marriages to close relatives are consistently reported in the more traditional rural areas and among the poorest and least educated in society [8]. Reports from some Arab countries have shown that consanguinity rates are lower in urban when compared to rural settings. Urban to rural first cousin rates in Algeria were 10% and 15% [14], in Egypt, 8.3% and 17.2% [15], and in Jordan, 29.8% and 37.9% [6], respectively. Likewise the mean inbreeding coefficient was lower in urban as compared to rural settings in Syria (0.0203 versus 0.0265) [16]. In Jordan, it was evident that the higher the level of education of the female partner, the lower the consanguinity rate. Only 12% of university educated females would marry their first cousins, whereas 25% of university educated males tend to marry first cousins [6]. Similar trends of lower consanguinity rates among educated women, but not educated men, were noticed in Yemen [17] and Tunisia [18].

On the other hand, social, religious, cultural, political and economic factors still play roles in favoring consanguineous marriages among the new generations just as strongly as they did among the older generations, particularly in rural areas. Consanguinity rates seem to be increasing at a higher pace in Qatar [19], Yemen [17], the United Arab Emirates (UAE) [20], and Tlemcen in Algeria [14]. In Morocco, a study indicated an increasing consanguinity rate from the previous (21.5%) to the present (25.4%) generation [21], while another study indicated a decreasing consanguinity rate [22]. Consanguinity rates are not declining in some Arab countries because it is generally accepted that the social advantages of consanguinity outweigh the disadvantages [23], and consanguinity is regarded as a deeply rooted cultural trend. It is believed that the practice of consanguinity has significant social and economic advantages. Consanguineous marriages among Arabs are respected because it is thought that they promote family stability, simplify financial premarital negotiations, offer a greater compatibility between the spouses and other family members, offer a lesser risk of hidden financial and health issues, and maintain the family land possessions [3,24,25]. Among 390 women attending reproductive health clinics in Jordan, consanguinity was protective against violence during pregnancy [26]. In all cases, reports on secular trends in consanguinity need to be treated with some caution because in countries where consanguinity is favored, major regional and ethnic differences in prevalence are commonly observed [3].

## Consanguinity and Reproductive Health

Research on the association of consanguinity with the different parameters of reproductive health in Arab countries is limited, both in quantity and in quality. Many studies fail to indicate clearly the different categories of consanguineous marriages in their methodology and thus the results are presented for consanguineous marriages as a single entity with the conclusions relying on a simple consanguineous versus non-consanguineous dichotomy. Given the wide range of F values in the 'consanguineous' group (F = 0.0156-0.125), with second cousin offspring (F = 0.0156) closer to non-consanguineous (F = 0) than to first cousins (F = 0.0625) or double first cousins (F = 0.125), such comparisons between consanguineous and non-consanguineous are thus not accurate. However, owing to the dearth of publications in the field among Arabs, this review will mention these studies with clear indication of the categories of consanguinity that are being compared.

## Negative Effects of Consanguinity on Reproductive Health

### Consanguinity and Congenital Malformations

Approximately 3-5% of all live newborns have a medically significant birth defect. The recent report by March of Dimes estimated birth defects to be >69.9/1000 live births in most Arab countries, as opposed to <52.1/1000 live births in Europe, North America and Australia [27]. Lower observed rates of 7.92/1000 births and 12.5/1000 births were registered in the UAE and Kuwait, respectively [28,29]. In Oman, among 21,988 births, 24.6 per 1000 births had major malformations [30]. Differences in birth defect rates in different countries and studies could be attributed to true differences among different populations or to different definitions of birth defects, different methods, and different time periods for ascertainment. The risk of birth defects in first-cousin marriages may be estimated to be 2-2.5 times the general population rate, mainly due to the expression of autosomal recessive disorders [23,31-33]. Another estimate puts the offspring of first cousin unions at a 1.7-2.8% increased risk for congenital defects above the population background risk [34]. However, these risk figures need validation for Arab countries through further well controlled evidence based and standardized research.

Frequency of consanguineous marriages was higher among parents of offspring with congenital malformations compared with the figures for the general population in all studies reported among Arabs, including in the UAE [28,35-37], Kuwait [29], Oman [30,38,39], Iraq [40,41], Jordan [42,43], Egypt [44], Lebanon [4,45], Tunisia [46], Arabs in Jerusalem [33], and Saudi Arabia [47]. After controlling for confounders, first cousin consanguinity remained significantly associated with an increased risk of congenital heart defects (CHD), where infants born to consanguineous parents had a higher risk of having a CHD diagnosed at birth compared to those born to unrelated parents in Lebanon [48,49], Saudi Arabia [47,50,51], Egypt [52], and Arabs in Israel [53]. Conversely, the overall incidence of CHD among 140,000 newborns in Oman, a country with high consanguinity rate, was similar to that reported from developed countries in Europe and America, insinuating that consanguinity is not a risk factor for CHD [54]. It could be argued, however, that although the overall incidence is not increased, the rates among consanguineous and non-consanguineous marriages may be different, a point that was not investigated in the study.

Consanguinity rates were noted to be higher among parents of newborns with congenital hydrocephalus [55] and neural tube defects [56,57] than in the general population in some studies, but not in others [58]. A positive association of consanguinity with cleft lip and/or palate was reported in the Palestinians [59], and the Lebanese [60], but not from studies in Kuwait and Saudi Arabia [61,62].

### Consanguinity and Postnatal Mortality

Countries with high rates of consanguineous marriages generally report smaller effects of consanguinity on mortality than populations with low rates of consanguineous marriages [63]. This finding is unsurprising, given the limited control for concomitant variables such as socioeconomic status, maternal education, birth intervals and public health facilities and practices in most consanguinity studies.

The most recent mortality estimate derived from a multinational study of over 600,000 pregnancies and live births, is that first cousin progeny experience 4.4% more pre-reproductive deaths than the offspring of non-consanguineous unions [64]. Most studies among Arabs have indicated that postnatal mortality is higher among offspring of consanguineous parents than among non-related parents [4,42,65-71]. Few studies have not detected this increase in postnatal mortality [35,68]. The increased postnatal mortality among the offspring of consanguineous parents may be related to the action of deleterious recessive genes and multi-gene complexes inherited from a common ancestor. The higher parity rate among consanguineous couples counterbalances the higher infant mortality; as a result, there may be equality in the number of living children among consanguineous and non-consanguineous couples.

### Consanguinity and Autosomal Recessive Disorders

In mathematical terms, consanguinity does not alter the allele frequencies of common disorders, but increases the probability of a mating between two individual heterozygotes for the same recessive mutant allele. In this regard, the risk for birth defects in the offspring of first-cousin marriage is expected to increase sharply compared to non-consanguineous marriages particularly for rare autosomal recessive disease genes, because for common recessive conditions, there is a high chance that the abnormal gene may be carried by unrelated spouses and may be expressed in their progeny.

In Arab populations and Diasporas, the deep-rooted norm of consanguineous marriage has been widely accused of being an important factor contributing to the preponderance of autosomal recessive genetic disorders [35,47,72-76]. In many parts of the Arab world, the society is still tribal. This has made the epidemiology of genetic disorders complicated, as many families and tribal groups are descended from a limited number of ancestors and some conditions are confined to specific villages, families, and tribal groups, leading to an unusual burden of genetic diseases in these communities [77]. Thus the extended family structure, commonly present in Arab societies and mostly associated with consanguinity, tends to display unique distribution patterns for genetic diseases that are not present in many other societies. There are disorders that are specifically prevalent among the Arabs, either uniformly or in certain locations, such as Bardet-Biedl syndrome, Meckel-Gruber syndrome, spinal muscular atrophy, osteopetrosis and renal tubular acidosis, Sanjad-Sakati syndrome, and congenital chloride diarrhea [78,79]. In an Arab society, mutation carriers mostly remain concentrated within the extended family and consanguineous marriages increase the probability of expression of autosomal recessive disorders when both mother and father are carriers of the mutation. Sometimes, autosomal recessive genes stay hidden within the family for generations and then show on the surface in a new consanguineous marriage within the family.

An analysis of data in the Catalogue for Transmission Genetics in Arabs (CTGA), a database on genetic disorders in Arab populations maintained by the Centre for Arab Genomic Studies, indicates that in contrast to international databases, the overwhelming proportion of the disorders in the CTGA Database follow a recessive mode of inheritance (63%) compared to the smaller proportion of dominantly inherited traits (27%). A detailed study of countries for which surveys on the occurrence of genetic disorders have been completed (United Arab Emirates, Bahrain, and Oman) indicates that recessive disorders are more in number than the dominant ones [80-82]. As explained above, given the high rates of consanguinity in these countries, this pattern is not entirely surprising. In a study from Jordan, the consanguinity rate among parents of affected with autosomal recessive conditions was around 85%, while it was 25-30% among parents of affected with other genetic conditions such as X-linked recessive, chromosomal and autosomal dominant [76].

## Neutral or Positive Effects of Consanguinity on Reproductive Health

Parallel to the huge body of literature detailing the negative effects of consanguinity on human health, there also exists a considerable amount of data that suggests that the practice of consanguinity is not the great evil that it is generally thought to be.

### Fetal Wastage

Multiple studies in highly consanguineous world populations have noted that fetal loss has no significant association with consanguinity. In Sudan, among 4,471 pregnancies, no significant difference in the reproductive loss was observed between the inbred and outbred groups [66]. In a study in Saudi Arabia, total prenatal losses were essentially the same among consanguineous and non-consanguineous couples [67]. Among 1867 married couples in Jordan, abortion rate was not affected by consanguinity [42]. Other studies have reported similar results [4,35,68,69,83-86]. Fewer studies noticed a higher rate of prenatal losses among consanguineous couples [13,70,87].

### Fertility

Consanguinity was generally not found to be associated with a significant positive or negative effect on fertility [83,88,89], although some international studies report a higher fertility among consanguineous couples [90,91]. Among Arabs, higher fertility rates and higher rates of live births were reported among first cousin couples than non-consanguineous couples in Qatar [87], Kuwait [92], Saudi Arabia [84], and Tunisia [69]. Similarly, in various ethnic groups from Mauritania (including: Soninkes, Poulard, Maures, Wolofs, and black Maures) consanguineous couples had averages of fertility significantly higher than those of non consanguineous couples [93]. Researchers tend to think that this increase in fertility could be a biological means of compensating for the increased risk of postnatal loss expected in related marriages or possibly to the earlier age at marriage, earlier first maternity and longer reproductive span among consanguineous as compared to non-consanguineous couples [88].

Effects of consanguineous marriages on couples' fertility and on offspring mortality were investigated in Beirut through a population-based health survey of 2,752 households. Total pregnancies, live births, and living children were significantly higher among consanguineous couples than among non-consanguineous ones, as was the proportion of dead among children ever born. However, no difference remained in either fertility or mortality, when allowance was made for socioeconomic status, religious affiliation, and marriage duration. The lack of significant pattern in the final analysis is interpreted as resulting from a long-term practice of consanguineous marriages [4].

Reports on the association of consanguinity with infertility are scarce among Arabs; a recent study from Lebanon pointed to a positive association between consanguinity and male factor infertility among 120 infertile males indicating the important contribution of recessive genetic factors to the etiology of male infertility [94].

### Consanguinity and Birth Anthropometric Measurements

Studies among Arabs related to the effect of consanguinity on anthropometric measurements such as birth weight gave conflicting results [84,95-99]. Studies from Jordan [43] and Arabs in Israel [85] detected a significant reduction in birth weight with consanguinity.

It seems that there is no definite correlation between consanguinity and anthropometric measurements in populations with high consanguinity rates. More studies using standardized methodology are recommended to verify any such correlation taking into consideration the changing socioeconomic and nutritional parameters among Arabs.

## Conclusive Remarks

Consanguineous marriages are widely practiced in several global populations, with some of the highest rates observed in the Arab World. Reports abound on both the negative and positive biological effects of consanguinity. In net terms, the reproductive criteria related to consanguineous versus non-consanguineous couples include earlier parental age at marriage, younger maternal age at first live birth, higher number of infants born to consanguineous parents, similar rates of abortions, and higher rates of postnatal mortality and birth defects in offspring of consanguineous parents. Furthermore, consanguineous unions lead to increased expression of autosomal recessive disorders. The CTGA Database on genetic disorders in Arab populations offers a clear evidence for a direct correlation between these two factors.

Studies on the association of consanguinity with chromosomal abnormalities such as Down syndrome and association with non-communicable disorders such as diabetes, hypertension, and psychiatric disorders among Arabs are presently non conclusive with the recommendation of performing standardized research in the future. Likewise, studies on the association of consanguinity with traits such as intelligence quotient and stature are scanty among Arabs and results of studies performed in Western countries cannot be applied directly to societies with high consanguinity rates such as the Arab society.

Scientifically, a considerable number of genes causing autosomal recessive conditions have been structurally and functionally determined at the molecular level through the joint collaboration of international and Arab scientists; these efforts should continue and expand given the high number of rare recessive disorders in the region.

Young Arabs contemplating marriage are nowadays seeking a scientifically sound answer to their questions: "Will our children be physically or mentally abnormal if I marry my cousin?" "How can we prevent having abnormal children?" Research on inbreeding is considered a priority in societies with high consanguinity rates to help understand and prevent the deleterious impact of consanguinity on health, and to provide standardized and evidence-based guidelines for health care providers to assist them in counseling for consanguinity.

## Conflicting interests

The authors declare that they have no competing interests.

## Authors' contributions

GOT: Initiated the concept of the paper, collected partial data on consanguinity (Table 1 and additional file 1), made the illustration used in the paper, supervised all the primary text authoring written by co-authors in Dubai.

PN: Authored the review on the positive aspects of consanguinity and collected partial data on consanguinity (Table 1 and additional file 1).

TO: Authored the review on the negative aspects of consanguinity and collected partial data on consanguinity (Table 1 and additional file 1).

MTA: Facilitated the collection of published data on consanguinity by offering services available at government medical bibliographic facilities and reviewed the final version of the manuscript.

NA: Discussed and approved the primary text of the manuscript as prepared by the team of the Centre for Arab Genomic Studies in Dubai.

HAH: Enriched the primary content of the paper co-authored in Dubai with extensively detailed data (Table 1 and additional file 1), and modified, revised, and added text content in different sections of the manuscript and made it reach to the present level.

## Supplementary Material

Additional file 1 Consanguinity rates in Arab populations. Click here for file

## References

[B1] Alwan A, Modell B (1997). Community control of genetic and congenital disorders.

[B2] Modell B, Darr A (2002). Science and society: genetic counselling and customary consanguineous marriage. Nat Rev Genet.

[B3] Bittles AH (2008). A community genetics perspective on consanguineous marriage. Community Genet.

[B4] Khlat M (1988). Consanguineous marriage and reproduction in Beirut, Lebanon. Am J Hum Genet.

[B5] Khlat M (1988). Consanguineous marriages in Beirut: time trends, spatial distribution. Soc Biol.

[B6] Khoury SA, Massad D (1992). Consanguineous marriage in Jordan. Am J Med Genet.

[B7] Vardi-Saliternik R, Friedlander Y, Cohen T (2002). Consanguinity in a population sample of Israeli Muslim Arabs, Christian Arabs and Druze. Ann Hum Biol.

[B8] Bittles AH, Grant JC, Shami SA (1993). Consanguinity as a determinant of reproductive behaviour and mortality in Pakistan. Int J Epidemiol.

[B9] Hamamy H, Jamhawi L, Al-Darawsheh J, Ajlouni K (2005). Consanguineous marriages in Jordan: why is the rate changing with time?. Clin Genet.

[B10] Al Arrayed SS (1995). The frequency of consanguineous marriages in the State of Bahrain. Bahrain Medical Bull.

[B11] Jaber L, Halpern GJ, Shohat T (2000). Trends in the frequencies of consanguineous marriages in the Israeli Arab community. Clin Genet.

[B12] Sharkia R, Zaid M, Athamna A, Cohen D, Azem A, Zalan A (2008). The changing pattern of consanguinity in a selected region of the Israeli Arab community. Am J Hum Biol.

[B13] Assaf S, Khawaja M (2009). Consanguinity trends and correlates in the Palestinian Territories. J Biosoc Sci.

[B14] Zaoui S, Biemont C (2002). [Frequency of consanguineous unions in the Tlemcen area (West Algeria)]. Sante.

[B15] Hafez M, El-Tahan H, Awadalla M, El-Khayat H, Abdel-Gafar A, Ghoneim M (1983). Consanguineous matings in the Egyptian population. J Med Genet.

[B16] Othman H, Saadat M (2009). Prevalence of consanguineous marriages in Syria. J Biosoc Sci.

[B17] Jurdi R, Saxena PC (2003). The prevalence and correlates of consanguineous marriages in Yemen: similarities and contrasts with other Arab countries. J Biosoc Sci.

[B18] Kerkeni E, Monastiri K, Saket B, Rudan D, Zgaga L, Ben CH (2006). Association among education level, occupation status, and consanguinity in Tunisia and Croatia. Croat Med J.

[B19] Bener A, Alali KA (2006). Consanguineous marriage in a newly developed country: the Qatari population. J Biosoc Sci.

[B20] al-Gazali LI, Bener A, Abdulrazzaq YM, Micallef R, al-Khayat AI, Gaber T (1997). Consanguineous marriages in the United Arab Emirates. J Biosoc Sci.

[B21] Talbi J, Khadmaoui AE, Soulaymani AEM, Chafik AEA (2007). Etude de la consanguinité dans la population marocaine. Impact sur le profil de la santé. Antropo.

[B22] Lamdouar BN (1994). [Consanguinity and public health in Morocco]. Bull Acad Natl Med.

[B23] Jaber L, Halpern GJ, Shohat M (1998). The impact of consanguinity worldwide. Community Genet.

[B24] Bittles A (2001). Consanguinity and its relevance to clinical genetics. Clin Genet.

[B25] Bittles AH (2002). Endogamy, consanguinity and community genetics. J Genet.

[B26] Clark CJ, Hill A, Jabbar K, Silverman JG (2009). Violence during pregnancy in Jordan: its prevalence and associated risk and protective factors. Violence Against Women.

[B27] Christianson A, Howson C, Modell B (2006). Global Report on Birth Defects The Hidden Toll of Dying and Disabled Children March of Dimes Birtn Defects Foundation.

[B28] Al Hosani H, Salah M, Abu-Zeid H, Farag HM, Saade D (2005). The National Congenital Anomalies Register in the United Arab Emirates. East Mediterr Health J.

[B29] Madi SA, Al-Naggar RL, Al-Awadi SA, Bastaki LA (2005). Profile of major congenital malformations in neonates in Al-Jahra region of Kuwait. East Mediterr Health J.

[B30] Sawardekar KP (2005). Profile of major congenital malformations at Nizwa Hospital, Oman: 10-year review. J Paediatr Child Health.

[B31] Stoltenberg C, Magnus P, Lie RT, Daltveit AK, Irgens LM (1997). Birth defects and parental consanguinity in Norway. Am J Epidemiol.

[B32] Zlotogora J (2002). What is the birth defect risk associated with consanguineous marriages?. Am J Med Genet.

[B33] Bromiker R, Glam-Baruch M, Gofin R, Hammerman C, Amitai Y (2004). Association of parental consanguinity with congenital malformations among Arab newborns in Jerusalem. Clin Genet.

[B34] Bennett R, Motulsky A, Bittles A, Hudgins L, Uhrich S, Doyle D, Silvey K, Scott R, Cheng E, McGillivray B, Steiner R, Olson D (2002). Genetic counseling and screening of consanguineous couples and their offspring: recommendations of the National Society of genetic Genetic Counselors. J of Genetic Counseling.

[B35] Abdulrazzaq YM, Bener A, al-Gazali LI, al-Khayat AI, Micallef R, Gaber T (1997). A study of possible deleterious effects of consanguinity. Clin Genet.

[B36] al-Gazali LI, Dawodu AH, Sabarinathan K, Varghese M (1995). The profile of major congenital abnormalities in the United Arab Emirates (UAE) population. J Med Genet.

[B37] Dawodu A, Al-Gazali L, Varady E, Varghese M, Nath K, Rajan V (2005). Genetic contribution to high neonatally lethal malformation rate in the United Arab Emirates. Community Genet.

[B38] Hasab A, Jaffer A (1997). Congenital anomalies among Omani births: a case control approach. Bull High Inst Public Health.

[B39] Patel PK (2007). Profile of major congenital anomalies in the Dhahira region, Oman. Ann Saudi Med.

[B40] Hamamy HA, al-Hakkak ZS (1989). Consanguinity and reproductive health in Iraq. Hum Hered.

[B41] Mahdi A (1992). Consanguinity and its effect on major congenital malformations. Iraqi Med J.

[B42] Khoury SA, Massad DF (2000). Consanguinity, fertility, reproductive wastage, infant mortality and congenital malformations in Jordan. Saudi Med J.

[B43] Obeidat BR, Khader YS, Amarin ZO, Kassawneh M, Al OM (2008). Consanguinity and Adverse Pregnancy Outcomes: The North of Jordan Experience. Matern Child Health J.

[B44] Temtamy SA, Abdel Meguid N, Mazen I, Ismail SR, Kassem NS, Bassiouni R (1998). A genetic epidemiological study of malformations at birth in Egypt. East Mediterr Health J.

[B45] Bittar Z (1998). Major congenital malformations presenting in the first 24 hours of life in 3865 consecutive births in south of Beirut. Incidence and pattern. J Med Liban.

[B46] Khrouf N, Spang R, Podgorna T, Miled SB, Moussaoui M, Chibani M (1986). Malformations in 10,000 consecutive births in Tunis. Acta Paediatr Scand.

[B47] El Mouzan MI, Al Salloum AA, Al Herbish AS, Qurachi MM, Al Omar AA (2008). Consanguinity and major genetic disorders in Saudi children: a community-based cross-sectional study. Ann Saudi Med.

[B48] Yunis K, Mumtaz G, Bitar F, Chamseddine F, Kassar M, Rashkidi J (2006). Consanguineous marriage and congenital heart defects: a case-control study in the neonatal period. Am J Med Genet A.

[B49] Nabulsi MM, Tamim H, Sabbagh M, Obeid MY, Yunis KA, Bitar FF (2003). Parental consanguinity and congenital heart malformations in a developing country. Am J Med Genet A.

[B50] Seliem MA, Bou-Holaigah IH, Al-Sannaa N (2007). Influence of consanguinity on the pattern of familial aggregation of congenital cardiovascular anomalies in an outpatient population: studies from the eastern province of Saudi Arabia. Community Genet.

[B51] Becker S, Al HZ (1999). First-cousin matings and congenital heart disease in Saudi Arabia. Community Genet.

[B52] Bassili A, Mokhtar SA, Dabous NI, Zaher SR, Mokhtar MM, Zaki A (2000). Congenital heart disease among school children in Alexandria, Egypt: an overview on prevalence and relative frequencies. J Trop Pediatr.

[B53] Gev D, Roguin N, Freundlich E (1986). Consanguinity and congenital heart disease in the rural Arab population in northern Israel. Hum Hered.

[B54] Subramanyan R, Joy J, Venugopalan P, Sapru A, al Khusaiby SM (2000). Incidence and spectrum of congenital heart disease in Oman. Ann Trop Paediatr.

[B55] Murshid WR, Jarallah JS, Dad MI (2000). Epidemiology of infantile hydrocephalus in Saudi Arabia: birth prevalence and associated factors. Pediatr Neurosurg.

[B56] Rajab A, Vaishnav A, Freeman NV, Patton MA (1998). Neural tube defects and congenital hydrocephalus in the Sultanate of Oman. J Trop Pediatr.

[B57] Murshid WR (2000). Spina bifida in Saudi Arabia: is consanguinity among the parents a risk factor?. Pediatr Neurosurg.

[B58] Asindi A, Shehri A (2001). Neural tube defects in the Asir region of Saudi Arabia. Ann Saudi Med.

[B59] Zlotogora J (1997). Genetic disorders among Palestinian Arabs: 1. Effects of consanguinity. Am J Med Genet.

[B60] Kanaan ZM, Mahfouz R, Tamim H (2008). The prevalence of consanguineous marriages in an underserved area in Lebanon and its association with congenital anomalies. Genet Test.

[B61] Aljohar A, Ravichandran K, Subhani S (2008). Pattern of cleft lip and palate in hospital-based population in Saudi Arabia: retrospective study. Cleft Palate Craniofac J.

[B62] al-Bustan SA, el-Zawahri MM, al-Adsani AM, Bang RL, Ghunaim I, Maher BS (2002). Epidemiological and genetic study of 121 cases of oral clefts in Kuwait. Orthod Craniofac Res.

[B63] Khlat M, Khoury M (1991). Inbreeding and diseases: demographic, genetic, and epidemiologic perspectives. Epidemiol Rev.

[B64] Bittles AH, Neel JV (1994). The costs of human inbreeding and their implications for variations at the DNA level. Nat Genet.

[B65] Wong SS, Anokute CC (1990). The effect of consanguinity on pregnancy outcome in Saudi Arabia. J R Soc Health.

[B66] Saha N, Hamad RE, Mohamed S (1990). Inbreeding effects on reproductive outcome in a Sudanese population. Hum Hered.

[B67] al Husain M, al Bunyan M (1997). Consanguineous marriages in a Saudi population and the effect of inbreeding on prenatal and postnatal mortality. Ann Trop Paediatr.

[B68] Al-Awadi SA, Naguib KK, Moussa MA, Farag TI, Teebi AS, el-Khalifa MY (1986). The effect of consanguineous marriages on reproductive wastage. Clin Genet.

[B69] Kerkeni E, Monastiri K, Saket B, Guediche MN, Ben CH (2007). Interplay of socio-economic factors, consanguinity, fertility, and offspring mortality in Monastir, Tunisia. Croat Med J.

[B70] Mokhtar MM, Abdel-Fattah MM (2001). Consanguinity and advanced maternal age as risk factors for reproductive losses in Alexandria, Egypt. Eur J Epidemiol.

[B71] Pedersen J (2002). The influence of consanguineous marriage on infant and child mortality among Palestinians in the West Bank and Gaza, Jordan, Lebanon and Syria. Community Genet.

[B72] el-Shafei A, Rao PS, Sandhu AK (1986). Congenital malformations and consanguinity. Aust N Z J Obstet Gynaecol.

[B73] Hoodfar E, Teebi AS (1996). Genetic referrals of Middle Eastern origin in a western city: inbreeding and disease profile. J Med Genet.

[B74] Mokhtar MM, Kotb SM, Ismail SR (1998). Autosomal recessive disorders among patients attending the genetic clinic in Alexandria. East Mediterr Health J.

[B75] Kerkeni E, Monastiri K, Saket B, Guediche MN, Ben CH (2007). Interplay of socio-economic factors, consanguinity, fertility, and offspring mortality in Monastir, Tunisia. Croat Med J.

[B76] Hamamy HA, Masri AT, Al-Hadidy AM, Ajlouni KM (2007). Consanguinity and genetic disorders. Profile from Jordan. Saudi Med J.

[B77] Al-Gazali L, Hamamy H, Al-Arrayad S (2006). Genetic disorders in the Arab world. BMJ.

[B78] Teebi AS, Teebi SA (2005). Genetic diversity among the Arabs. Community Genet.

[B79] Teebi A, Farag T (1997). Genetic Disorders Among Arab Populations Oxford Monographs on Medical Genetics No30.

[B80] Tadmouri GO, Tadmouri GO, Taleb Al Ali M, Al Khaja N (2004). Genetic disorders in Arab Populations.

[B81] Tadmouri GO, Tadmouri GO, Taleb Al Ali M, Al Khaja N (2006). Genetic disorders in Arab Populations: A 2006 Update.

[B82] Tadmouri GO, Tadmouri GO, Taleb Al Ali M, Al Khaja N (2008). Genetic disorders in Arab Populations: A 2008 Update.

[B83] Saad FA, Jauniaux E (2002). Recurrent early pregnancy loss and consanguinity. Reprod Biomed Online.

[B84] al-Abdulkareem AA, Ballal SG (1998). Consanguineous marriage in an urban area of Saudi Arabia: rates and adverse health effects on the offspring. J Community Health.

[B85] Jaber L, Merlob P, Gabriel R, Shohat M (1997). Effects of consanguineous marriage on reproductive outcome in an Arab community in Israel. J Med Genet.

[B86] Hamamy H, Al-Bayati N, Al-Kubaisy W (1986). Consanguineous marriages in Iraqi urban population and the effect on pregnancy outcome and infant mortality. Iraqi Med J.

[B87] Bener A, Hussain R (2006). Consanguineous unions and child health in the State of Qatar. Paediatr Perinat Epidemiol.

[B88] Bittles AH, Grant JC, Sullivan SG, Hussain R (2002). Does inbreeding lead to decreased human fertility?. Ann Hum Biol.

[B89] Hussain R, Bittles AH (2004). Assessment of association between consanguinity and fertility in Asian populations. J Health Popul Nutr.

[B90] Fuster V (2003). Inbreeding pattern and reproductive success in a rural community from Galicia (Spain). J Biosoc Sci.

[B91] Helgason A, Palsson S, Gudbjartsson DF, Kristjansson T, Stefansson K (2008). An association between the kinship and fertility of human couples. Science.

[B92] Kandari Y (2007). Fertility and its relationship with sociocultural factors in Kuwaiti society. East Mediterr Health J.

[B93] Hammami A, Chalbi N, Ben AM, Elgazzeh M (2005). [Effects of consanguinity and social factors on mortality and fertility in Mauritania]. Tunis Med.

[B94] Inhorn MC, Kobeissi L, Nassar Z, Lakkis D, Fakih MH (2009). Consanguinity and family clustering of male factor infertility in Lebanon. Fertil Steril.

[B95] al-Eissa YA, Ba'Aqeel HS, Haque KN (1991). Low birthweight in Riyadh, Saudi Arabia: incidence and risk factors. Ann Trop Paediatr.

[B96] Al-Sekait MA (1989). Maternal influences on birth weight. J R Soc Health.

[B97] Khlat M (1989). Inbreeding effects on fetal growth in Beirut, Lebanon. Am J Phys Anthropol.

[B98] Mumtaz G, Tamim H, Kanaan M, Khawaja M, Khogali M, Wakim G (2007). Effect of consanguinity on birth weight for gestational age in a developing country. Am J Epidemiol.

[B99] Saedi-Wong S, al-Frayh AR (1989). Effects of consanguineous matings on anthropometric measurements of Saudi newborn infants. Fam Pract.

[B100] Benallegue A, Kedji F (1984). [Consanguinity and public health. Algerian study]. Arch Fr Pediatr.

[B101] al-Arrayed SS (1999). Review of the spectrum of genetic diseases in Bahrain. East Mediterr Health J.

[B102] Stevenson AC, Johnston HA, Stewart MI, Golding DR (1966). Congenital malformations. A report of a study of series of consecutive births in 24 centres. Bull World Health Organ.

[B103] Mohamed MS (1995). An epidemiological study on consanguineous marriage among urban population in Alexandria. J Egypt Public Health Assoc.

[B104] Habib Z, Book JA (1983). Consanguinity and incidence of thalassaemia in Egypt. Hereditas.

[B105] Hussien FH (1971). Endogamy in Egyptian Nubia. J Biosoc Sci.

[B106] Badr FM (1972). Genetic studies of Egyptian Nubian populations. I. Frequency and types of consanguineous marriages. Hum Hered.

[B107] Hamamy HA, al-Hakkak ZS (1989). Consanguinity and reproductive health in Iraq. Hum Hered.

[B108] Ali MM, Shah IH (2000). Sanctions and childhood mortality in Iraq. Lancet.

[B109] COSIT (Central Organization for Statistics and Information Technology) (2005). Iraq Living Conditions Survey 2004: Analytical Report.

[B110] Cook R, Hanslip A (1966). Mortality among offspring of consanguineous marriage in a rural area of East Jordan. J Trop Pediatr Afr Child Health.

[B111] al-Salem M, Rawashdeh N (1993). Consanguinity in north Jordan: prevalence and pattern. J Biosoc Sci.

[B112] Nabulsi A (1995). Mating patterns of the Abbad tribe in Jordan. Soc Biol.

[B113] Sueyoshi S, Ohtsuka R (2003). Effects of polygyny and consanguinity on high fertility in the rural Arab population in South Jordan. J Biosoc Sci.

[B114] El-Alfi OS, Bahig AH, Abdul Salam T, Shaath R (1969). Birth weights in Kuwait, and their relation to consanguinity and to birth order. J Kuwait Medical Assoc.

[B115] Al-Awadi SA, Moussa MA, Naguib KK, Farag TI, Teebi AS, el-Khalifa M (1985). Consanguinity among the Kuwaiti population. Clin Genet.

[B116] Al-Kandari YY (2007). Fertility and its relationship with sociocultural factors in Kuwaiti society. East Mediterr Health J.

[B117] Radovanovic Z, Shah N, Behbehani J (1999). Prevalence and social correlates to consanguinity in Kuwait. Ann Saudi Med.

[B118] Khlat M, Halabi S, Khudr A, Der Kaloustian VM (1986). Perception of consanguineous marriages and their genetic effects among a sample of couples from Beirut. Am J Med Genet.

[B119] Tamim H, Khogali M, Beydoun H, Melki I, Yunis K (2003). Consanguinity and apnea of prematurity. Am J Epidemiol.

[B120] Barbour B, Salameh P (2009). Consanguinity in Lebanon: prevalence, distribution and determinants. J Biosoc Sci.

[B121] Broadhead RC, Sehgal KC (1981). Consanguinity and congenital anomalies in East Libya. Garyounis Med J.

[B122] Baali A (1994). Etude anthropobiologique d'une population berbere semi-isolee du Hgaut-Atlas: Valle d'Azgour, cercle d'Amizmiz, Marrakech. PhD Thesis.

[B123] Lamdouar BN (1994). [Consanguinity and public health in Morocco]. Bull Acad Natl Med.

[B124] Cherkaoui M, Baali A, Larrouy G, Sevin A, Boetsch G (2005). Consanguinity, fertility of couples and mortality of children in the high Atlas population (commons of Anougal and Azgour, Marrakesh, Morroco). Intl J Anthropol.

[B125] Rajab A, Patton MA (2000). A study of consanguinity in the Sultanate of Oman. Ann Hum Biol.

[B126] Freundlich E, Hino N (1984). Consanguineous marriage among rural Arabs in Israel. Isr J Med Sci.

[B127] Jaber L, Bailey-Wilson JE, Haj-Yehia M, Hernandez J, Shohat M (1994). Consanguineous matings in an Israeli-Arab community. Arch Pediatr Adolesc Med.

[B128] Zlotogora J, Habiballa H, Odatalla A, Barges S (2002). Changing family structure in a modernizing society: a study of marriage patterns in a single Muslim village in Israel. Am J Hum Biol.

[B129] Bashi J (1977). Effects of inbreeding on cognitive performance. Nature.

[B130] El-Hazmi MA, al-Swailem AR, Warsy AS, al-Swailem AM, Sulaimani R, al-Meshari AA (1995). Consanguinity among the Saudi Arabian population. J Med Genet.

[B131] El-Mouzan MI, Al-Salloum AA, Al-Herbish AS, Qurachi MM, Al-Omar AA (2007). Regional variations in the prevalence of consanguinity in Saudi Arabia. Saudi Med J.

[B132] Ahmed AH (1979). Consanguinity and schizophrenia in Sudan. Br J Psychiatry.

[B133] Saha N, el Sheikh FS (1988). Inbreeding levels in Khartoum. J Biosoc Sci.

[B134] Prothro ET, Diab LN (1974). Changing family patterns in the Arab East.

[B135] Riou S, el YC, Chaabouni H (1989). [Consanguinity in the population of northern Tunisia]. Tunis Med.

[B136] Ben AS, Masmoudi S, Beltaief N, Hachicha S, Ayadi H (2004). Consanguinity and endogamy in Northern Tunisia and its impact on non-syndromic deafness. Genet Epidemiol.

[B137] Fahmy NA, Benson PF, Al-Garrah DB (1993). Consanguinity in UAE: Prevalence and analysis of some risk factors. Emirates Med J.

[B138] Gunaid AA, Hummad NA, Tamim KA (2004). Consanguineous marriage in the capital city Sana'a, Yemen. J Biosoc Sci.

[B139] Bittles AH The global prevalence of consanguinity.

