# Supplementary material for: Consanguinity and reproductive health among Arabs
Source: Reprod Health. 2009 Oct 8;6:17. doi: 10.1186/1742-4755-6-17 (PMC2765422; doi:10.1186/1742-4755-6-17)
Supplement: Additional file 1 — Consanguinity rates in Arab populations. [file 1742-4755-6-17-S1.DOC]

**Additional File 1.** Consanguinity rates in Arab populations.

| **Country** | **Location** | **Collection Period*** | **Sample Size** | >1C **,1C*** | **Overall*** | **Reference** |
| --- | --- | --- | --- | --- | --- | --- |
| **Algeria** |  | 1979 | 120,491 |  | 22.6 | [100] |
|  | Tlemcen | 2002 [?] | 3,983 | 11.3 | 34 [?] | [14] |
| **Bahrain** |  | 1995 (Past generation) | 100 |  | 45.5 [?] | [10] |
|  |  | 1995 (Present generation) | 500 |  | 39.4 [?] | [10] |
|  |  | 1999 | 1,000 | 24.5 | 43.1 | [101] |
| **Egypt** | Alexandria | 1961-1964 | 9,475 |  | 32.8 [>1C,1C,2C,<2C] | [102] |
|  | Nubia | 1965-1967 | 281 | 39 | 60.5 | [105] |
|  | All Nubia | 1967-1968 | 1,782 | 41.5-47.2 | 72.9-80.4 | [106] |
|  |  | 1970s | 505 | 23.2 | 23.2 | [140] |
|  |  | 1983 [?] | 26,554 | 14.3 | 29 [>1C,1C,2C,<2C] | [15] |
|  | Alexandria | 1995 [?] | 500 | 15.8 | 22.8 [?] | [103] |
|  | Alexandria | 1998-2000 | 2,081 | 15.9 | 20.9 [?] | [70] |
| **Iraq** | Baghdad | 1986 | 4,491 | 29.2 | 57.8 [1C,1.5C,2C,<2C] | [86] |
|  | Baghdad | 1989 | 382 | 29-32.2 | 47.1-57.8 | [107] |
|  | Central and South Iraq | 1999 | 23,105 |  | 60 [?] | [108] |
|  | North Kurdish Region | 1999 | 14,035 |  | 47 [?] | [108] |
|  | All Iraq | 2004 | 23,937 | 33 | 33 | [109] |
| **Jordan** | Irbid (Rural) | 1963-1964 | 1,097 | 39 | 52.1 | [110] |
|  |  | 1969-1979 | 1,989 | 32.8 | 50.3 [1C,1.5C,2C,<2C] | [6] |
|  | Balqa | 1986-1989 | 340 |  | 40.3 | [141] |
|  | Irbid | 1993 [?] | 900 | 37.3 | 63.7 [1C,1.5C,2C,<2C] | [142] |
|  | South Ghor | 2003 [?] | 608 |  | 58.1 [?] | [143] |
|  | Amman | 2004 (present generation) | 2,594 | 19.5 | 28.5 [1C,1.5C,2C,<2C] | [9] |
|  | North of Jordan | 2007 | 3,269 |  | 49 [>1C,1C,2C,<2C] | [43] |
| **Kuwait** |  | 1969 [?] | 2,133 |  | 38.4 [?] | [114] |
|  |  | 1983 | 5,007 | 30.2 | 64.3 [1C,1.5C,2C,<2C] | [144] |
|  | Jahra | 1996-1997 | 555 | 31.7 | 42.2 | [145] |
|  | Kuwait City | 1996-1997 | 404 | 16.9 | 22.5 | [145] |
|  |  | 2002 | 7315 |  | 54.8 [?] | [146] |
| **Lebanon** | Beirut | 1981-1982 | 750 |  | 26 | [118] |
|  | Beirut | 1983-1984 | 2,854 | 14 | 25 [1C,1.5C,2C,<2C] | [4] |
|  | Beirut | 1983-1984 | 3,033 |  | 25 [?] | [5] |
|  | Beirut | 1998-2001 | 21,723 | 6.7 | 12.8 [1C,2C,<2C] | [119] |
|  | Beqaa | 2007 [?] | 552 | 31 | 42 [?] | [60] |
|  |  | 2009 [?] | 1,556 (women) | 31.6 | 37.8 [1C,1.5C,2C,<2C] | [120] |
| **Libya** | Benghazi | 1981 [?] | <500 |  | 48.4 [?] | [147] |
| **Mauritania** |  | 2005 [?] | 2,413 |  | 47.2 [?] | [93] |
| **Morocco** | High-Atlas Valleys | 1904-1985 |  |  | 23.1 [?] | [122] |
|  |  | 1940 | 582 | 10 | 21.5 | [21] |
|  |  | 1984 | 291 | 8.6 | 25.4 | [21] |
|  | All Morocco | 1982-1992 | 4,773 |  | 19.9 | [123] |
|  | High-Atlas Valleys | 2005 [?] | 438 |  | 28 [?] | [148] |
| **Oman** |  | 1994-1997 | 60,895 | 24.1 | 56.3 [1C,1.5C,2C,<2C] | [125] |
| **Palestine** |  | 1977 | 3,203 | 34.2 | 34.2 | [129] |
|  | Western Galilee | 1976-1983 | 1,546 |  | 32.2 | [53] |
|  | Western Galilee | 1984 | 550 |  | 39 | [149] |
|  |  | 1980-1985 (Past generation) |  | 23.9 | 33.1 [?] | [12] |
|  |  | 2000-2004 (Present generation) |  | 13.6 | 25.9 [?] | [12] |
|  |  | 1981-1985 | 3,328 | 23.5 | 40.6 | [11] |
|  | Muslim | 1990-1992 | 1,025 |  | 32.1 | [7] |
|  | Bedouin | 1990-1992 | 278 |  | 45.3 | [7]l |
|  | Christian | 1990-1992 | 107 |  | 20.6 | [7] |
|  | Druze | 1990-1992 | 115 |  | 40.9 | [7] |
|  |  | 1992 | 8,521 (total) | 25.3 | 44.3 [?] | [150] |
|  |  |  | 5,098 (rural) |  | 30.6 | [150] |
|  |  |  | 2,267 (suburban) |  | 28.2 | [150] |
|  |  |  | 1,156 (urban) |  | 24.2 | [150] |
|  | West Bank | 1995 | 10,409 | 27.2 | 66.3 [>1C,1C,2C,<2C] | [71] |
|  | Gaza | 1995 | 5,778 | 31.6 | 64.5 [>1C,1C,2C,<2C] | [71] |
|  | Palestinians in Jordan | 1996 | 4,950 | 26.1 | 47.1 [>1C,1C,2C,<2C] | [71] |
|  | Palestinians in Lebanon | 1998 | 3,972 | 19.8 | 32.3 [>1C,1C,2C,<2C] | [71] |
|  | Palestinians in Jordan | 1999 | 2,888 | 26 | 46.9 [>1C,1C,2C,<2C] | [71] |
|  | Palestinians in Syria | 2001 | 4,195 | 18.3 | 30.5 [>1C,1C,2C,<2C] | [71] |
|  | Lower Galilee | 1970-2000 | 483 | 17.5 | 17.5 [1C] | [128] |
|  |  | 1995 (Past generation) | 16,171 | 29.2 | 49.8 [>1C,1C,2C,<2C] | [13] |
|  |  | 2004 (Present generation) | 4,971 | 27.7 | 45.4 [>1C,1C,2C,<2C] | [13] |
| **Qatar** | Doha | 2004 | 1,515 | 34.8 | 54 [>1C,1C,2C,<2C] | [19] |
| **Saudi Arabia** | Riyadh | 1983-1986 | 4,497 | 31 | 54.3 [?] | [99] |
|  | Riyadh | 1993 | 2,001 | 28.4 | 51.1 [1C,1.5C,2C,<2C] | [67] |
|  |  | 1995 [?] | 3,212 | 25.8 | 56.8 [1C,1.5C,2C,<2C] | [130] |
|  | Dammam | 1998 [?] | 1,307 | 39.3 | 52 [1C,1.5C,2C,<2C] | [84] |
|  | Al-Baha | 2004-2005 | 487 | 29 | 42.1 [?] | [131] |
|  | Al-Jouf | 2004-2005 | 593 | 34.8 | 53.5 [?] | [131] |
|  | Assir | 2004-2005 | 833 | 24.6 | 44.5 [?] | [131] |
|  | Eastern Province | 2004-2005 | 1,032 | 33.3 | 57.8 [?] | [131] |
|  | Gizan | 2004-2005 | 565 | 33 | 53.5 [?] | [131] |
|  | Hail | 2004-2005 | 505 | 25.1 | 48.9 [?] | [131] |
|  | Madinah | 2004-2005 | 618 | 39.2 | 67.2 [?] | [131] |
|  | Makkah | 2004-2005 | 2,278 | 32.4 | 55.9 [?] | [131] |
|  | Najran | 2004-2005 | 472 | 28.4 | 66.7 [?] | [131] |
|  | Northern Borders | 2004-2005 | 504 | 31.4 | 63.9 [?] | [131] |
|  | Qassim | 2004-2005 | 713 | 29.6 | 46.7 [?] | [131] |
|  | Riyadh | 2004-2005 | 2,522 | 42.3 | 60 [?] | [131] |
|  | Tabuk | 2004-2005 | 432 | 28.3 | 60 [?] | [131] |
|  | All Saudi Arabia | 2004-2005 | 11,554 | 33.6 | 56 [?] | [131] |
| **Sudan** | Gezira | 1969-1974 | 2,999 | 44.2 | 44.2 | [132] |
|  | Khartoum | 1988 [?] | 4,833 |  | 52 | [133] |
|  | Khartoum | 1990 [?] | 926 | 49.5 | 63.3 | [66] |
| **Syria** | Urban  Rural  All Syria | 1974 [?]  2009  2009  2009 | 36,574  31,384  67,958 | 28.7 | 33 [?]  30.3 [>1C 1C,1.5C,2C,<2C]  39.8 [>1C 1C,1.5C,2C,<2C]  35.4 [>1C 1C,1.5C,2C,<2C] | [134]  [16]  [16]  [16] |
| **Tunisia** | Northern Tunisia | 1983-1985 | 5,767 | 23 | 26.9 | [135] |
|  | Monastir | 1989-1990 | 1,741 | 17.4 | 24.8 | [69] |
|  | Monastir | 2003-2004 | 1,016 |  | 20.1 | [18] |
|  | Bizerte | 2000-2002 | 4,860 | 20.8 | 39.33 [1C,2C,<2C] | [136] |
| **UAE** |  | 1993 [?] |  |  | 51 [?] | [137] |
|  | Al Ain | 1992-1994 | 16,419 [?] |  | 54 [?] | [36] |
|  | Al Ain | 1994-1995 | 1,502 | 28.2 | 54.2 [1C,1.5C,2C,<2C] | [20] |
|  | Dubai | 1994-1995 | 531 | 20.7 | 40 [1C,1.5C,2C,<2C] | [20] |
|  | Al-Ain & Dubai |  | 2,033 | 26.2 | 50.5 [1C,2C,<2C] | [20] |
| **Yemen** |  | 1997 | 9,762 | 34 | 40 [?] | [17] |
|  | Sanaa | 2000 | 1,050 | 32 | 44.7 | [138] |

Abbreviations: [?] = Unknown year of sampling or unknown types of consanguineous marriages; [>1C] = Double first-cousin marriage; [1C] = First-cousin marriage; [<1C] = Marriage beyond first-cousins; [1.5C] = First-cousin once removed marriage; [2C] = Second-cousin marriage; [<2C] = Marriage between distant relatives beyond second-cousins.
